# Antibacterial activities of the methanol extracts of seven Cameroonian dietary plants against bacteria expressing MDR phenotypes

**DOI:** 10.1186/2193-1801-2-363

**Published:** 2013-07-31

**Authors:** Jackson A Seukep, Aimé G Fankam, Doriane E Djeussi, Igor K Voukeng, Simplice B Tankeo, Jaurès AK Noumdem, Antoine HLN Kuete, Victor Kuete

**Affiliations:** Department of Biochemistry, Faculty of Science, University of Dschang, Dschang, Cameroon; Department of Organic Chemistry, Faculty of Science, University of Dschang, Dschang, Cameroon

**Keywords:** Antibacterial activity, Cameroon, Dietary plants, Efflux pumps, Gram-negative bacteria, Multi-drug resistant

## Abstract

The morbidity and mortality caused by bacterial infections significantly increased with resistance to commonly used antibiotics. This is partially due to the activation of efflux pumps in Gram-negative bacteria. The present work designed to assess the *in vitro* antibacterial activities of seven Cameroonian dietary plants (*Sesamum indicum, Sesamum radiatum, Cinnamomum zeylanicum, Corchous olitorius, Cyperus esculentus, Adansonia digitata, Aframomum kayserianum*)*,* against multidrug resistant (MDR) Gram-negative bacteria over expressing active efflux pumps. The standard phytochemical methods were used to detect the main classes of secondary metabolites in the extracts. The antibacterial activities of the studied extracts in the absence or presence of an efflux pump inhibitor (PAβN) were evaluated using liquid microbroth dilution method. The results obtained indicated that apart from the extract of *C. esculentus*, all other samples contained alkaloids, phenols and polyphenols meanwhile other classes of chemicals were selectively present. The studied extracts displayed antibacterial activities with minimal inhibitory concentrations (MICs) values ranged from 64 to 1024 μg/mL on the majority of the 27 tested microbial strains. The extract of *S. indicum* was active against 77.77% of the tested microorganisms whilst the lowest MIC value (64 μg/mL) was recorded with that of *A. kayserianum* against *E. aerogenes* EA294. The results of the present work provide baseline information on the possible used of the tested Cameroonian dietary plants in the treatment of bacterial infections including multi-drug resistant phenotypes.

## Introduction

The continuous emergence of multidrug-resistant (MDR) bacteria drastically reduces the efficacy of our antibiotic armory, and consequently increases the frequency of therapeutic failure Falagas and Bliziotis (2007). The resistance of bacteria to chemically unrelated antimicrobial agents may be associated with the over-expression of efflux pumps (Poole 2004 Li and Nikaido 2009). In Gram-negative bacteria, many of these efflux pumps belong to the resistance-nodulation-cell division (RND) family of tripartite efflux pumps. Among those efflux pumps, those belonging to the AcrAB-TolC family are detected in many clinical bacterial isolates and are reported to be a key factor in the expression of the MDR phenotypes (Mallea et al. 2003; Lomovskaya et al. 2004). This efflux pumps mechanism can be blocked by various inhibitors which restore the intracellular concentration as well as the activities of the antibiotics (Chollet et al. 2004; Pagès and Amaral 2009). The scarcity of original synthetic antibiotics has stimulated the search for new antibacterial agents from medicinal plants. This explains our endeavor to evaluate *in vitro* the antibacterial activities of Cameroonian dietary plants namely the beans of *Sesamum indicum,* the stem and leaves of *Sesamum radiatum, Corchous olitorius* and *Cyperus esculentus,* the bark of *Cinnamomum zeylanicum,* the fruits of *Adansonia digitata* and *Aframomum kayserianum* against Gram-negative bacteria expressing MDR phenotypes. The role of efflux pumps in the activity of our plants extracts was also investigated using a previously described efflux pump inhibitor.

## Material and methods

### Plant materials and extraction

The seven edible plants used in this work were purchased from Bafoussam local market, West Region of Cameroon in January 2012. The collected plant samples were the beans of *Sesamum indicum,* the stem and leaves of *Sesamum radiatum, Corchous olitorius* and *Cyperus esculentus,* the bark of *Cinnamomum zeylanicum,* the fruits of *Adansonia digitata* and *Aframomum kayserianum*. The plants were further identified at the National Herbarium (Yaoundé, Cameroon) where voucher specimens were deposited under a reference number (Table 1). The powdered air-dried sample from each plant was extracted with methanol for 48 h at room temperature. The extract was then concentrated under reduced pressure to give a residue that constituted the crude extract. They were then kept under 4°C until further use.

**Table 1:** **Plants used in the present study and evidence of their activities**

Plants samples, part used and herbarium voucher number^a^	Traditional used	Known antimicrobial activities of plants
Bombaceae *Adansonia digitata* (Fruits) 42417/HNC	Febrifuge, antidysentery, antioxydant, analgesic, antidiarrheal (Kaboré et al. 2011); immunostimulant, hepatoprotective (Al-Qarawi et al. 2010); anti-small pox, anti-rubella (Wickens 1979).	Aqueous, ethanol and petroleum ether extracts **Q** : Ec (Yagoub 2008). Antiviral activity against Vi, Vhs (Vimalanathan and Hudson 2009), Vp (Anani et al. 2000).
Cyperaceae *Cyperus esculentus* (Fruits)14977/SRFC	(/)^b^	(/)
Pedaliaceae *Sesamum indicum* (Beans) 42898/HNC	Liniment, laxative, emollient (Kokate et al. 2005); lenitif, anticonstipation, anti-carries (Awobajo et al. 2009)**,** antitumor (Xu et al. 2010), hepatoprotective (Kumar et al. 2011), hypoglycemic (Nakano and Kwak 2006).	(/)
Lauraceae *Cinnamomum zeylanicum.* (Bark) Blume 22309/SRFC	Digestive disorders, anti dysentery, laxative.	Isolated compounds (Cinnamaldehyde and Eugenol) from essential oil showed an activity: against Pl (Gende et al. 2008) ; antibacterial activities against multi-resistant Gram-negative bacteria (Pa, PS, Kp, EC., Ea, Ecl) (Voukeng et al. 2012)
Tiliaceae *Corchous olitorius* (Stem and leaves) 14725/SRFC	Gonorrhoea, cystitis chronic, analgesic, febrifuge, antitumor, anti-inflammatory (Zacharia et al. 2006; Ramadevi and Ganapaty 2011), diuretic, cardiotonic (Ramadevi and Ganapaty 2011).	Methanol, chloroform and petroleum ether extracts. **S**: Bs, Pa, Sm. **F**: Sa, Ec, La, ML, San, Sau, Kp. **M:** Ea, Pv. **Q**: Rs, Ca (Ramadevi and Ganapaty 2011), Ec Zacharia et al. (2006) Sau Bp, St, Sb, Ss, Sd,Pa, Kp, Ec,Vc Pal et al. (2006).
Pedaliaceae *Sesamum radiatum* Schum et Thom (Stem and leaves) 8797/SRFC	Antirhume, anticatarrate, against ocular pains and cutaneous eruptions (Bankole et al. 2007), antimicrobial (Shittu et al. 2007; Osibote et al. 2010)**.**	Methanol and ethanol extracts **S** : Ca (Shittu et al. 2007). Essential oil :**Q:** Csp. Ec, Ec ATCC 25922, Kp, Pm, Pa, Sal, Sa, Sm Tane et al. (2005).
Zingiberaceae *Aframomum kayserianum* (Fruits) K.schum 18884/SRFC	Anti-mumps, dysmenorrhoeas, vermifuge (Tane et al. 2005).	Isolated compound Aframodial from this plant showed an activity: **S :** Sc, Sp,Ha, Cu, Scl, Pc.; **M :** Mm, Rc, An, Sau, Bs. **F :** Ec, Pa (Ayafor et al. 1994)**.**

^*a*^*(HNC)*, Cameroon National Herbarium; *(SRF)*, Société des reserves forestière du Cameroun; ^*b*^*(/)*. Not reported. Screened Activity: Significant (S:MIC < 100 μg/mL), moderate (M: 100 < MIC ≤ 625 μg/mL), Weak (F:MIC > 625 μg/mL) (Kuete 2010), *Q*, qualitative activity based on the determination of the inhibition zone. *Ca Candida albicans, St Salmonella typhi*, *An Aspergilus nicfer*, *Bs Bacillus subtilis*, *EC Escherichia coli*, *Kp Klebsiella pneumoniae*, *Pa Pseudomonas aeruginosa*, *Pvt Proteus vulgaris*, *Sau Staphylococcus aureus*, *Cu Candida utilis*, *Sc Saccharomyces cereviciae*, *Sm Streptococcus mutans*, *Sa Streptococcus aeoginosa*, *La Lactobacillus acidophillus*, *ML Micrococcus luteus*, *San Streptococcus anginosus*, *Ea Enterobacter aerogenes*, *Rs Rhizoctonia solanic*, *Bp Bacillus punilus*, *Sb Shigella boydii*, *Ss Shigella sonnei*, *Sd Shigella dysenteria*, *Vc Vibrio cholerae*, *Csp Citrobacter sp*, *Pm Proteus mirabilis*, *Sal Staphylococcus albus*, *Sma Serratia marcescens*, *Sp Schizosaccharomyces pombe*, *Ha Hansenula anomala*, *Scl Sclerotinia libertiana*, *PC Penicillim crustasum*, *Mm Mucor mucedo*, *Rc Rhizopus chinensis*, *Ecl Enterobacter cloacae*, *PS Providencia stuartii*, *Pl Paenibacillus larvae***,***Vhs* herpes simplex virus,*Vp* Virus of poliomyelitis, *VI* Influenza. Virus.

### Preliminary phytochemical investigations

The presence of major secondary metabolite classes, namely, alkaloids, flavonoids, phenols, saponins, tannins, anthocyanins, anthraquinones, sterol, and triterpenes (Table 2) was determined using common phytochemical methods Harbone (1973).

**Table 2:** **Bacterial strains and features**

Bacteria	Features	References
*Escherichia coli*		
**ATCC8739**	Reference strain of *Escherichia coli*	
**ATCC10536**	Reference strain of *Escherichia coli*	
**W3110**	Wild-type E. coli K-12	(Bagliomi et al. 2003)
**MC4100**	*Wild*-*type E*. *coli K*-*12* , KAN^R^	(Martina 2002)
**AG100A**	AG100 ΔacrAB::KAN^R^	(Monks et al. 1992; Okusu et al. 1996; Pradel and Pagès 2002)
**AG100Atet**	ΔacrAB mutant AG100, owing acrF gene markedly overexpressed; TET^R^	Monks et al. (1992)
**AG102**	ΔacrAB mutant AG100	Chevalier et al. (2000)
**AG100**	Wild-type E. coli K-12	Lorenzi et al. (2009)
*E*. *aerugenes*		
**ATCC13048**	Reference strain	
**EA294**	EA 289 ΔacrAB: KAN^R^	(Pradel and Pagès 2002; Ghisalberti et al. 2005)
**CM64**	CHLR resistant variant obtained from ATCC13048 over-expressing the AcrAB pump	Ghisalberti et al. (2005)
**EA298**	EA 289 tolC::KAN^R^	(Pradel and Pagès 2002; Ghisalberti et al. 2005)
**EA27**	Clinical MDR isolate exhibiting energy-dependent norfloxacin and chloramphenicol efflux with KAN^R^ and AMP^R^ and NAL^R^ and STR^R^ and TET^R^	(Mallèa et al. 2002; Ghisalberti et al. 2005)
**EA289**	KAN sensitive derivative of EA27	Ghisalberti et al. (2005)
*Klebsiella pneumoniae*		
**ATCC11296**	Reference strain	
**Kp55**	Clinical MDR isolate, TET^R^ ,AMP^R^ ,ATM^R^ , and CEF^R^	Chevalier et al. (2000)
**Kp63**	Clinical MDR isolate, TET^R^,CHL^R^,AMP^R^, and ATM^R^	Fredrickson et al. (2004)
**K2**	*Klebsiella pneumonia* AcrAB-TolC	Laboratory collection
**K24**	*Klebsiella pneumonia*e AcrAB-Tolc	
***Pseudomonas aeruginosa***		
**PA01**	Reference strain	
**PA124**	MDR clinical isolate	Lorenzi et al. (2009)
*Providencia stuartii*		
**ATCC29916**	Reference strain	
**PS2636**	Clinical MDR isolate, AcrAB	Laboratory collection
**PS299645**	Clinical MDR isolate, AcrAB	
*Enterobacter cloacae*		
**BM47**	*Enterobacter cloacae* AcrAB-Tolc	
**ECCI69**	*Enterobacter cloacae* AcrAB-TolC	
**BM67**	*Enterobacter cloacae* AcrAB-TolC	

AMP^R^, ATM^R^, CEF^R^, CFT^R^, CHL^R^, FEP^R^, KAN^R^, MOX^R^, NAL^R^, NOR^R^ STR^R^, and TET^R^ Resistance to ampicillin, aztreonam, cephalothin, cefadroxil, chloramphenicol, cefepime, kanamycin, moxalactam, streptomycin, and tetracycline; *MDR* multidrug resistant., *OMPF and OMPC* Outer Membran Protein F and C respectively. *AcrAB-TolC* efflux pump AcrAB associate to TolC porine, *Pa Pseudomonas aeruginosa*.

### Bacteria strains and culture media

The studied microorganisms included references (from the American Type Culture Collection) and clinical (Laboratory collection) strains of *Escherichia coli, Enterobacter aerogenes, Providencia stuartii, Pseudomonas aeruginosa, Klebsiella pneumoniae,* and *Enterobacter cloacae* (Table 2). They were maintained on agar slant at 4°C and sub-cultured on a fresh appropriate agar plates 24 h prior to any antimicrobial test. Mueller Hinton Agar (MHA) was used for the activation of bacteria and the Mueller Hinton Broth (MHB) was used for the MIC determinations.

### Bacterial susceptibility determinations

The respective MICs of samples on the studied bacteria were determined by using rapid INT colorimetric assay Mativandlela et al. (2006). Briefly, the test samples were first dissolved in DMSO/MHB. The solution obtained was then added to MHB, and serially diluted two fold (in a 96-well microplate). One hundred microlitres (100 μL) of inoculum (1.5 × 10^6^ CFU/mL) prepared in MHB was then added. The plates were covered with a sterile plate sealer, then agitated to mix the contents of the wells using a shaker and incubated at 37°C for 18 hrs. Wells containing MHB, 100 μL of inoculum and DMSO at a final concentration of 2.5% served as negative control (this internal control was systematically added). Chloramphenicol (CHL) was used as reference antibiotic. The MICs of samples were detected after 18 hrs of incubation at 37°C, following addition (40 μL) of 0.2 mg/mL INT and incubation at 37°C for 30 min Kuete et al. (2008). Viable bacteria reduced the yellow dye to pink. MIC was defined as the lowest sample concentration that prevented this change and exhibited complete inhibition of bacterial growth. For the determination of MBC, he microplates ones were filled by 150 μL of MHB without extract of plant; for wells not having received a INT (during the reading of the MIC), 50 μL of the contents of the wells corresponding to the concentrations higher or equal to the MIC was taken and introduced into these microplates. These were then incubated during 48 h à 37°C followed by revelation with the INT. All the concentrations to which we did not observe pink coloring were taken as bactericides and smallest of those, was noted as MBC. Samples were tested alone and then, in the presence of PAβN at 30 μg/mL final concentration.

## Results

### Phytochemical composition of the plant extracts

The results of qualitative analysis showed that all the crude extracts tested, except that for *C. esculentus*, contained alkaloids, phenols and polyphenols; others phytochemicals classes being selectively detected (Table 3).

**Table 3:** **Parts used, extraction yields, physical aspect and phytochemical composition of the plant extracts**

Extracts	***S***. ***radiatum***	***C***. ***zeylanicum***	***C***. ***olitorius***	***S***. ***indicum***	***A***. ***kayserianum***	***A***. ***digitata***	***C***. ***esculentus***
Parts used	Stem and leaves	Bark	Stem and leaves	Beans	Fruits	Fruits	Fruits
Yield* (%)	6.67	5.65	4.81	4.87	4.95	3.40	15.21
Alkaloids	+	+	+	+	+	+	-
Anthocyanins	-	+	-	-	+	-	-
Anthraquinones	-	-	-	+	-	-	-
Flavonoids	+	-	-	-	-	+	-
Phenols	+	+	+	+	+	+	-
Tannins	+	-	+	-	+	-	-
Triterpenes	-	+	-	+	+	+	+
Sterols	+	-	+	+	-	+	+
Saponins	-	-	+	+	+	+	+

(+): Present; (−): Absent; * yield calculated as the ratio of the mass of the obtained methanol extract/mass of the plant powder.

### Antibacterial activity of the plant extracts

The various strains and MDR isolates were tested for their susceptibilities to plants extracts and reference antibiotic (chloramphenicol). Chloramphenicol was also tested the presence of PAßN, a well-known efflux pump inhibitor to confirm the role of efflux pumps in the resistance of the studied microorganisms. Assays were performed using the broth microdilution method. The results depicted in Table 4 indicate that the plant extracts exhibited activities depending of bacteria strains, with MICs values ranged from 64 to 1024 μg/mL on the majority of the 27 tested microbial strains. At the tested concentration range (8 to 1024 μg/mL), the extracts which displayed activities against the majority of bacteria strains were those from *S. indicum* (active against 77.77% of the tested bacteria), *C. zeylanicum* (70.37%), *S. radiatum* (66.66%), *C. olitorius* (62.96%), *A. kayserianum* (51.85%), *C. esculentus* (18,52%) and *A. digitata* (14,81%). The lowest MIC value (64 μg/mL) was recorded with the extract of *A. kayserianum* against *E. aerogenes* EA294. Other extracts exhibited weak activities against a limited number of strains studied. A keen look of the results of Table 4 also shows that the extract of *S. indicum* displayed the best spectrum of bactericidal effect with a ratio MBC/MIC ≤ 4 on 6 bacterial strains, followed by that of *S. radiatum* (2/27) and those of *C zeylanicum, C. olitorius, A. kayserianum* (1/27). Only the extract of *A. digitata* did not present any bactericidal activity.

**Table 4:** **MIC, MBC and MBC/MIC ratios of plants extracts and CHL on the studied bacterial species**

Tested bacteria	Extracts and antimicrobial parameters (MIC and MBC in μg/mL)								
	***S***. ***radiatum***	***C***. ***zeylanicum***	***C***. ***olitorius***	***S***. ***indicum***	***A***. ***kayserianum***	***A***. ***digitata***	***C***. ***esculentus***	CHL	CHL + PβBN
*E*. *coli*									
**ATCC8739**	1024 (−)	-	-	-	-	-	-	8 (512)	4
**ATCC10536**	512 (−)	-	1024 (−)	512 (−)	-	512 (−)	512 (512)	4 (128)	1
**W 3110**	-	1024 (−)	-	1024 (−)	512 (−)	-	-	4 (32)	1
**MC4100**	-	512 (512-)	1024 (−)	-	1024 (−)	-	-	128 (512)	8
**AG100 A**	-	512 (−)	128 (512)	512 (512)	1024 (−)	-	256 (−)	4 (64)	0.5
**AG100Atet**	1024 (−)	512 (−)	1024 (−)	512 (−)	512 (−)	-	1024 (−)	64 (8)	8
**AG102**	1024 (−)	512 (−)	1024 (−)	1024 (−)	1024 (−)	-	-	32 (512)	8
**AG100**	-	1024 (−)	512 (−)	512 (−)	-	-	-	4(128)	<4
***E*.*aerogenes***									
**ATCC13048**	-	-	512 (−)	1024 (−)	-	-	-	8 (32)	4
**EA294**	-	**256** (−)	-	-	**64** (512)	-	-	32 (512)	8
**CM64**	1024 (−)	1024(−)	- (−)	1024 (−)	-	-	-	128 (−)	64
**EA298**	1024 (−)	512 (−)	1024 (−)	1024 (1024)	1024 (−)	256 (−)	512 (−)	256 (−)	64
**EA27**	-	-	1024 (−)	**256** (1024)	512 (−)	-	-	-	32
**EA289**	1024 (−)	1024 (−)	-	1024 (−)	-	-	-	256 (−)	64
***K*.*pneumoniae***									
**ATCC11296**	512 (−)	**256** (−)	-	512 (−)	-	-	-	8 (256)	4
**KP55**	512 (−)	512 (−)	1024 (−)	512 (512)	1024 (1024)	-	-	4 (128)	1
**KP63**	1024 (−)	1024 (−)	1024 (−)	1024 (−)	1024 (−)	-	-	128 (−)	32
**K2**	1024 (−)	1024 (−)	512 (−)	**256** (256)	-	1024 (−)	-	4 (128)	1
**K24**	-	512 (−)	1024 (−)	**256** (−)	512 (−)	-	-	32 (512)	32
***P*.*aeruginosa***									
**PA01**	1024 (−)	-	-	-	-	-	-	32 (256)	8
**PA124**	1024 (−)	-	-	-	-	-	-	64 (512)	16
***P*.*stuartii***									
**ATCC29916**	**256** (1024)	**256** (−)	-	-	512 (−)	-	-	4 (32)	2
**PS2636**	1024 (−)	-	512 (−)	512 (1024)	1024 (−)	-	-	4 (64)	2
**PS299645**	-	512 (−)	1024 (−)	1024 (−)	-	256 (−)	1024 (−)	16 (64)	4
***E*.*cloacae***									
**BM47**	1024 (−)	1024 (−)	1024 (−)	1024 (−)	-	-	-	256 (−)	16
**ECCI69**	1024 (−)	-	512 (−)	1024 (−)	-	-	-	256 (−)	16
**BM67**	512 512	512 (−)	-	1024 (−)	1024 (−)	-	-	512 (−)	16

**(−)**:> 1024 μg/mL for the extracts and > 512 μg/mL for chloramphenicol and non given MBC/MIC. NT: not tested. (): MBC in μg/m.

### Role of efflux pumps in susceptibility of Gram-negative bacteria to the tested plants extracts

The various strains and MDR isolates were also tested for their susceptibility to CHL in the presence of PAβN. As shown in Table 4, PAβN improved the activity of CHL to all studied bacteria.

## Discussion

The extract from *A. digitata,* others extracts exhibited antibacterial activities against at least one of the tested bacteria. The differences in antibacterial activities were noted between the various extracts and could be related to the differences in their phytochemical composition as shown in Table 2. Except in the extract of *C. esculentus,* the alkaloids, phenols and polyphenols were detected in all extracts. The antibacterial activities of many molecules belonging to these classes of compounds were shown (Cowan 1999; Kuete et al. 2009). Sharma and Singh (2011) also associated the antibacterial activities of the medicinal plants to the presence of flavonoids, tannins and alkaloids. It should however be mentioned that the detection of the bioactive phytochemical classes in a plant is not a guarantee for any biological property, as this will depend on the types of compounds, as well as their concentrations and possible interaction with other constituents.

Alkaloids, phenols, sterols, polyphenols, triterpenes, anthraquinones and saponins were detected in the extract of *S. indicum*. The presence of the anthocyanins and the flavonoids in this plant was previously reported Awobajo et al. (2009), though such classes of chemicals were not detected in this work. This could be explained by fact that the presence of the secondary metabolites in a plant depend on the environmental factors such as climate, chemical nature of the ground in which plant grow, the period of harvest, conditions of drying and extraction method Bruneton (1999). To the best of our knowledge, no antibacterial activity of this plant was shown up to now, but presence of the various classes of secondary metabolites could explain its activity on majority of tested strains obtained in this work. In addition the antibacterial activities of the essential oil of *S. radiatum* (Shittu et al. 2006 Shittu et al. 2007; Konan et al. 2008).

Voukeng and collaborators (2012) documented the antibacterial activities of methanol extract of the sheets of *C. zeylanicum* against MDR Gram-negative bacteria used in this work. The presence of the phenolic compounds detected in thebark of this plant could explain its activities. Significantantibacterial activities of the methanol extract of *C. olitorius* were shown against the Gram-positive bacteria Pal et al. (2006). Also, the roots of this plant showed a significant antibacterial activity Ramadevi and Ganapaty (2011). The antibacterial activities of this plant as shown in this work provide therefore additional data on its antimicrobial potentials. Plants of the genus *Aframomum* are known for their antibacterial activities, and this has been assigned to the presence of terpenoids such as aframodial Ayafor et al. (1994). This could also explain the activity observed with the extract of *A. kayserianum* in the present work. Several former studies showed the presence of the terpenoids, phenolic and alkaloids in the extract of *A. digitata* (Wickens 1979; Chadare et al. 2009). The aqueous, ethanol, and petroleum-ether extracts of this plant have shown an antibacterial activity against *E. coli* Yagoub (2008). However, we observed a weak antibacterial activity of this plant. The extract of *C. esculentus* contains less secondary classes of metabolites. Only sterols, triterpenes and saponins were detected. To the best of our knowledge, the antibacterial of this plant is being reported for the first time. Generally, the weak antibacterial activity observed with the majority of the extracts could be explained by the fact that bacterial strains used are MDR phenotypes expressing active efflux pumps Cattoir (2004), as shown with the increase of the activity of CHL in the presence of PAβN (Table 4). It was demonstrated that efflux pumps decrease the intracellular concentration of chemicals and consequently their activities (Bambeke and Pagès 2010; Kuete 2010). These efflux pumps can be blocked in a competitive way or not, by an efflux inhibitor, which restore therefore not only intracellular concentration, but also the activity of antibiotics (Pagès and Amaral 2009; Kuete 2010). Finally, the results obtained with the studied plants are encouraging not only because we are dealing with MDR bacteria, but also the fact investigated plant materials are food plants which are relatively non toxic.

## Conclusion

The results of the present work provide baseline information on the possible used of the tested Cameroonian dietary plants in the treatment of bacterial infections including MDR phenotypes.
